# O Polissacarídeo de Astragalus Melhora a Biogênese e a Função Mitocondrial em Cardiomiócitos através da Ativação da AMPK

**DOI:** 10.36660/abc.20250854

**Published:** 2026-09-18

**Authors:** Chengbin Wang, Shuhan Lv, Xingde Liu

**Affiliations:** 1 Guizhou University of Traditional Chinese Medicine Department of Second Clinical Medical College Guiyang China Guizhou University of Traditional Chinese Medicine – Department of Second Clinical Medical College, Guiyang – China

**Keywords:** Miócitos Cardíacos, Biogênese de Organelas, Insuficiência Cardíaca

## Abstract

**Fundamento:**

A disfunção mitocondrial é um fator chave na insuficiência cardíaca, o que reforça a necessidade de terapias que promovam a biogênese e a função mitocondrial. O polissacarídeo de Astragalus (APS), um composto bioativo extraído da *Astragalus membranaceus*, demonstrou propriedades cardioprotetoras, mas seus efeitos diretos na homeostase mitocondrial em cardiomiócitos ainda não estão claros.

**Objetivos:**

Este estudo teve como objetivo investigar os efeitos do APS na função mitocondrial e na biogênese em cardiomiócitos HL-1 e elucidar o envolvimento da via de sinalização da proteína quinase ativada por AMP (AMPK)/coativador 1-alfa do receptor gama ativado por proliferador de peroxissoma (PGC-1α).

**Métodos:**

Cardiomiócitos HL-1 foram tratados com concentrações não tóxicas de APS (1,25-2,5 g/L) por 48 horas. A respiração mitocondrial foi avaliada por meio do analisador Seahorse XF, os níveis de adenosina trifosfato (ATP) foram quantificados e a atividade do Complexo IV foi mensurada. A biogênese mitocondrial foi avaliada pela razão DNA mitocondrial (mtDNA)/DNA nuclear (nDNA), níveis de citocromo B e massa mitocondrial. Proteínas e genes-chave, incluindo PGC-1α, fator respiratório nuclear 2 (NRF2), fator de transcrição mitocondrial A (TFAM), proteína desacopladora 2 (UCP2) e AMPK fosforilada (p-AMPK), foram analisados por Western blot e qPCR. O inibidor de AMPK, Composto C, foi utilizado para confirmar o papel da via.

**Resultados:**

O tratamento com APS aumentou significativamente a respiração mitocondrial, a produção de ATP e a atividade do Complexo IV. Promoveu a biogênese mitocondrial, evidenciada pelo aumento da razão mtDNA/nDNA e da massa mitocondrial. O APS aumentou a expressão de PGC-1α, NRF2, TFAM e UCP2, e ativou a fosforilação da AMPK. Todos esses efeitos benéficos foram abolidos pela inibição da AMPK com o Composto C.

**Conclusões:**

O APS melhora a biogênese e a função mitocondrial em cardiomiócitos através da ativação da via de sinalização AMPK/PGC-1α, identificando-o como um potencial agente terapêutico para doenças cardíacas associadas à disfunção mitocondrial.

## Graphical abstract

**Figura Central:**
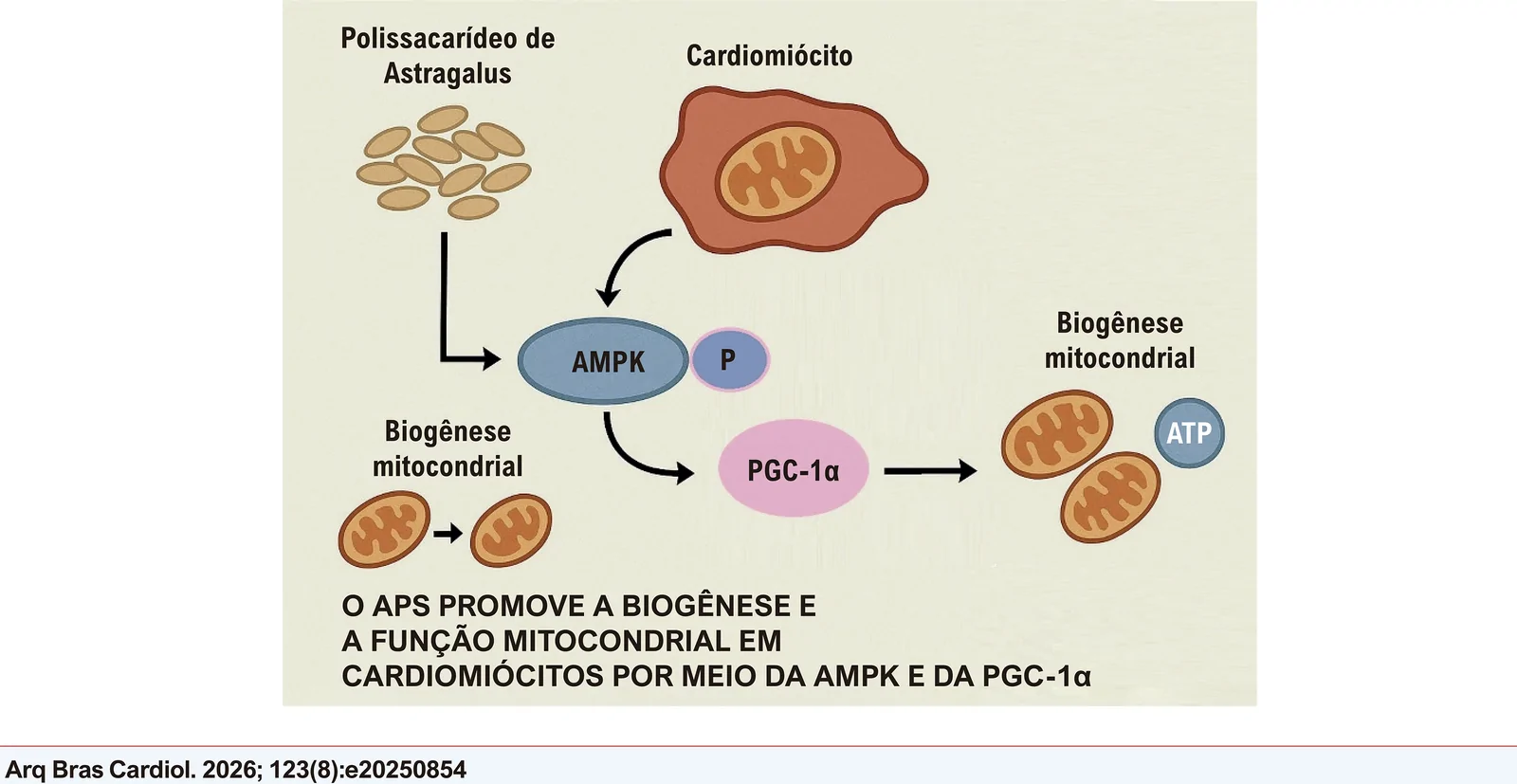
O Polissacarídeo de Astragalus Melhora a Biogênese e a Função Mitocondrial em Cardiomiócitos através da Ativação da AMPK

## Introdução

A disfunção mitocondrial é uma característica marcante de diversas doenças cardiovasculares, incluindo insuficiência cardíaca, cardiomiopatia isquêmica e cardiomiopatia diabética. Nos cardiomiócitos, que possuem uma das maiores demandas energéticas entre os tipos celulares, a integridade mitocondrial é essencial para a manutenção da função contrátil, da homeostase do cálcio e do equilíbrio redox.^1-3^ A disfunção na fosforilação oxidativa mitocondrial leva à depleção de adenosina trifosfato (ATP), à produção excessiva de espécies reativas de oxigênio (ROS) e à morte eventual dos cardiomiócitos, contribuindo assim para a progressão da insuficiência cardíaca.^4,5^ Para compensar danos ou perda mitocondrial, as células ativam a biogênese mitocondrial — um processo rigorosamente regulado que envolve a replicação do DNA mitocondrial (mtDNA), a expansão da massa mitocondrial e a regulação positiva de proteínas mitocondriais codificadas pelo núcleo.^6-9^ Fundamental para esse processo é o coativador 1-alfa do receptor gama ativado por proliferadores de peroxissoma (PGC-1α), um coativador transcricional que orquestra a expressão gênica mitocondrial por meio da interação com fatores respiratórios nucleares (NRFs) e o fator de transcrição mitocondrial A (TFAM).^10,11^ Contudo, em condições patológicas, esse mecanismo adaptativo é frequentemente atenuado, levando à insuficiência mitocondrial persistente. Portanto, estratégias farmacológicas que restauram ou aprimoram a biogênese mitocondrial mediada por PGC-1α têm surgido como abordagens terapêuticas promissoras para cardiomiopatias mitocondriais.

O polissacarídeo de Astragalus (APS), um dos principais componentes bioativos da erva medicinal tradicional chinesa *Astragalus membranaceus*, tem atraído considerável atenção por suas propriedades cardioprotetoras, antioxidantes e anti-inflamatórias.^12-15^ Foi relatado que o APS melhora a lesão miocárdica em modelos de isquemia-reperfusão, cardiomiopatia diabética e doença cardíaca hipertensiva. Diversos estudos demonstraram que o APS modula vias de sinalização celular, incluindo a fosfoinositídeo 3-quinase (PI3K)/proteína quinase B (Akt), o fator nuclear kappa-B (NF-κB) e o fator de crescimento transformador beta 1 (TGF-β1), sugerindo seu potencial regulatório pleiotrópico.^16-19^ Evidências crescentes destacam o potencial de produtos naturais, incluindo flavonoides, saponinas e polissacarídeos, para modular a função e a biogênese mitocondrial em doenças cardiovasculares. Revisões recentes enfatizaram que compostos naturais podem atuar na dinâmica mitocondrial, reduzir o estresse oxidativo e regular a mitofagia, exercendo, assim, efeitos cardioprotetores.^20,21^ Em particular, compostos derivados de plantas, como a berberina, o resveratrol e vários extratos de ervas, demonstraram ativar a via de sinalização da proteína quinase ativada por AMP (AMPK)/PGC-1α, promovendo a biogênese mitocondrial e melhorando a função cardíaca.^22^ No entanto, seu papel preciso na biologia mitocondrial, particularmente em relação à biogênese mitocondrial e ao metabolismo energético em cardiomiócitos, permanece em grande parte inexplorado. Dado o papel crítico da homeostase mitocondrial na função cardíaca, hipotetizamos que o APS promove a biogênese e a função mitocondrial em cardiomiócitos por meio da ativação da via de sinalização AMPK/PGC-1α. Para testar essa hipótese, investigamos os efeitos do APS na função e biogênese mitocondrial em cardiomiócitos HL-1 e elucidamos os mecanismos moleculares subjacentes (Figura Central).

## Materiais e Métodos

### Cultura e tratamento celular

Cardiomiócitos murinos HL-1 (Millipore, EUA) foram cultivados em meio Claycomb suplementado com 10% de soro fetal bovino (SFB), 100 U/mL de penicilina, 100 μg/mL de estreptomicina e 0,1 mM de norepinefrina (Sigma-Aldrich, EUA). As células foram mantidas em uma incubadora umidificada a 37 °C com 5% de CO_2_. Para o tratamento, o APS (pureza ≥98%, Yuanye Bio-Technology, China) foi dissolvido em água estéril para preparar uma solução estoque de 100 g/L, que foi então filtrada através de um filtro estéril de 0,22 μm e diluída nas concentrações indicadas (0–250 g/L) com meio de cultura imediatamente antes do uso. As células foram tratadas por 48 horas. Em experimentos selecionados, o composto C (MedChemExpress, EUA) foi dissolvido em dimetilsulfóxido (DMSO) para preparar uma solução estoque de 10 mM e armazenado a −20 °C. Imediatamente antes do uso, a solução estoque foi diluída em meio de cultura até uma concentração final de 10 μM (com uma concentração final de DMSO <0,1%). As células foram pré-tratadas com o composto C por 1 hora antes da administração de APS.

### Ensaio CCK-8

A viabilidade celular foi avaliada utilizando o kit de contagem celular CCK-8 (Dojindo, Japão), seguindo as instruções do fabricante. Células HL-1 foram semeadas em placas de 96 poços (1 × 10^4^ células/poço) e tratadas com APS nas concentrações de 0, 0,25, 0,5, 1,25, 2,5, 5, 25, 50 e 250 g/L por 48 horas. Após o tratamento, 10 μL do reagente CCK-8 foram adicionados a cada poço e incubados por 2 horas a 37°C no escuro. A absorbância foi medida a 450 nm usando um leitor de microplacas (BioTek, EUA).

### Liberação de LDH

A atividade da lactato desidrogenase (LDH) no sobrenadante da cultura foi medida utilizando um kit comercial de ensaio de citotoxicidade de LDH (Beyotime, China). de acordo com as instruções do fabricante, conforme descrito anteriormente para a avaliação da citotoxicidade.^23^Após o tratamento, 100 μL do meio de cultura foram coletados de cada poço e a atividade da LDH foi determinada espectrofotometricamente a 490 nm. Os resultados foram expressos em U/L.

### Atividade do Complexo IV

A atividade da citocromo c oxidase (Complexo IV) foi medida utilizando um kit de ensaio colorimétrico de atividade enzimática (Solarbio, China), seguindo o protocolo do fabricante, conforme descrito anteriormente.^24^ As células HL-1 foram lisadas e a atividade enzimática foi quantificada a 550 nm. Os dados foram normalizados pelo conteúdo proteico e expressos em nmol/min/mg de proteína.

### Medição da função respiratória mitocondrial

A respiração mitocondrial foi avaliada utilizando um analisador Seahorse XFe24 (Agilent Technologies, EUA). As taxas de consumo de oxigênio (OCR) basal e máxima foram medidas utilizando o kit Seahorse XF Cell Mito Stress Test, conforme descrito anteriormente, com injeção sequencial de oligomicina (1 μM), carbonil cianeto-4-(trifluorometoxi)fenil-hidrazona (FCCP, 1 μM) e rotenona/antimicina A (0,5 μM cada).^25^ Todos os compostos foram dissolvidos de acordo com as instruções do fabricante: oligomicina e FCCP foram dissolvidos em DMSO para preparar soluções estoque de 10 mM, enquanto rotenona e antimicina A foram dissolvidas em DMSO para preparar soluções estoque de 5 mM. As soluções estoque foram aliquotadas e armazenadas a −20 °C, sendo diluídas para as concentrações de trabalho em meio de ensaio Seahorse imediatamente antes do uso. Células HL-1 foram semeadas em placas Seahorse e tratadas com APS (1,25 ou 2,5 g/L) por 48 horas antes da medição. Os valores de OCR foram normalizados pela concentração de proteína.

### Determinação da produção de ATP

Os níveis intracelulares de ATP foram quantificados utilizando um kit de ensaio de ATP (Beyotime, China) baseado em uma reação de bioluminescência catalisada por luciferase, conforme descrito anteriormente.^26^As células foram lisadas em tampão de lise com ATP e a intensidade da luminescência foi medida usando um luminômetro (Promega, EUA). As concentrações de proteína foram determinadas utilizando o ensaio de ácido bicinconínico (BCA) para normalização. O conteúdo de ATP foi normalizado para proteína total e expresso em nmol/mg de proteína.

### Quantificação da razão entre mtDNA e nDNA

O DNA total foi extraído utilizando o kit TIANamp Genomic DNA (Tiangen, China). O conteúdo relativo de mtDNA foi quantificado por PCR em tempo real utilizando primers direcionados ao gene mitocondrial do citocromo B e ao gene nuclear da β-actina. A razão mtDNA/nDNA foi calculada utilizando o método 2^⁻Ct^.

### Coloração com MitoTracker Red

A massa mitocondrial foi avaliada utilizando MitoTracker Red CMXRos (Thermo Fisher Scientific, EUA). O corante MitoTracker Red foi dissolvido em DMSO anidro de alta pureza para preparar uma solução estoque de 1 mM, aliquotada e armazenada a −20 °C, protegida da luz. Imediatamente antes do uso, a solução estoque foi diluída em meio de cultura sem soro, previamente aquecido, até uma concentração final de 100 nM. As células foram incubadas com a solução de coloração por 30 minutos a 37 °C, no escuro, lavadas duas vezes com PBS e visualizadas utilizando um microscópio de fluorescência (Olympus IX73, Japão). Para a análise quantitativa, a intensidade de fluorescência foi medida por citometria de fluxo (BD FACSCanto II).

### PCR em tempo real (qPCR)

O RNA total foi extraído utilizando o reagente TRIzol (Invitrogen, EUA) e a síntese de cDNA foi realizada utilizando um kit de reagentes PrimeScript RT (Takara, Japão). A qPCR foi realizada com SYBR Green Master Mix (Roche, Suíça) em um sistema LightCycler 480. As condições de ciclagem térmica foram as seguintes: pré-incubação inicial a 95 °C por 10 minutos, seguida por 40 ciclos de desnaturação a 95 °C por 15 segundos, anelamento a 60 °C por 30 segundos e extensão a 72 °C por 30 segundos. Uma análise da curva de fusão foi realizada ao final de cada corrida para verificar a especificidade da amplificação. Os níveis de expressão gênica de PGC-1α, NRF2, TFAM, UCP2 e citocromo B foram normalizados para gliceraldeído-3-fosfato desidrogenase (GAPDH) utilizando o método 2⁻ΔΔCt. As sequências dos primers estão listadas na Tabela Suplementar S1.

### Análise de Western blot

A proteína total foi extraída com tampão de lise RIPA (*radioimmunoprecipitation assay*) contendo inibidores de protease e fosfatase (Beyotime, China). Quantidades iguais de proteína (30 μg) foram separadas por eletroforese em gel de poliacrilamida com dodecil sulfato de sódio (SDS-PAGE) utilizando géis de 12% e transferidas para membranas de fluoreto de polivinilideno (PVDF) (Millipore, EUA). As membranas foram bloqueadas em leite desnatado a 5% durante 1 hora à temperatura ambiente e, em seguida, incubadas durante a noite a 4 °C com anticorpos primários contra PGC-1α, NRF2, TFAM, UCP2, AMPKα fosforilada (Thr172), ACC fosforilada (Ser79) e β-actina (todos da Cell Signaling Technology, EUA). Anticorpos secundários conjugados com HRP foram utilizados e as bandas foram visualizadas por quimioluminescência aprimorada (ECL, Thermo Fisher) com tempos de exposição de 30 segundos a 2 minutos, dependendo da intensidade do sinal. As bandas de proteína foram quantificadas por densitometria usando o software ImageJ (National Institutes of Health, EUA), com as intensidades de sinal normalizadas em relação aos controles de carregamento de β-actina. Os resultados foram expressos como variação relativa ao grupo controle. As informações sobre os anticorpos estão listadas na Tabela Suplementar S2.

### Análise estatística

Todos os dados são expressos como média ± desvio padrão (DP) de pelo menos três experimentos independentes. A normalidade da distribuição dos dados foi testada utilizando o teste de Shapiro-Wilk. Para experimentos envolvendo um único fator, a significância estatística foi avaliada por meio de análise de variância (ANOVA) de uma via, seguida pelo teste post hoc de Tukey para comparações múltiplas. Para experimentos envolvendo dois fatores, foi realizada ANOVA de duas vias para avaliar os efeitos principais e as interações, seguida pelo teste de comparações múltiplas de Tukey. Um valor de p < 0,05 foi considerado estatisticamente significativo. As análises foram realizadas utilizando o GraphPad Prism 9.0 (GraphPad Software, EUA).

## Resultados

### O APS apresenta baixa citotoxicidade em baixas concentrações

Antes de avaliar os efeitos do APS na função mitocondrial, avaliamos primeiramente sua citotoxicidade em cardiomiócitos HL-1.As células foram expostas a concentrações crescentes de APS (0–250 g/L) durante 48 horas. Conforme demonstrado pelo ensaio CCK-8, baixas concentrações de APS (1,25–2,5 g/L) não afetaram significativamente a viabilidade celular em comparação com o controle (Figura 1A), enquanto altas concentrações (≥25 g/L) levaram a uma diminuição significativa e dose-dependente da viabilidade. De forma consistente, os ensaios de liberação de LDH revelaram citotoxicidade mínima em 1,25 e 2,5 g/L, mas a atividade da LDH aumentou acentuadamente em concentrações mais elevadas (Figura 1B). Esses dados sugerem que o APS é bem tolerado em baixas concentrações e pode exercer efeitos citoprotetores em condições não tóxicas.

**Figura 1:**
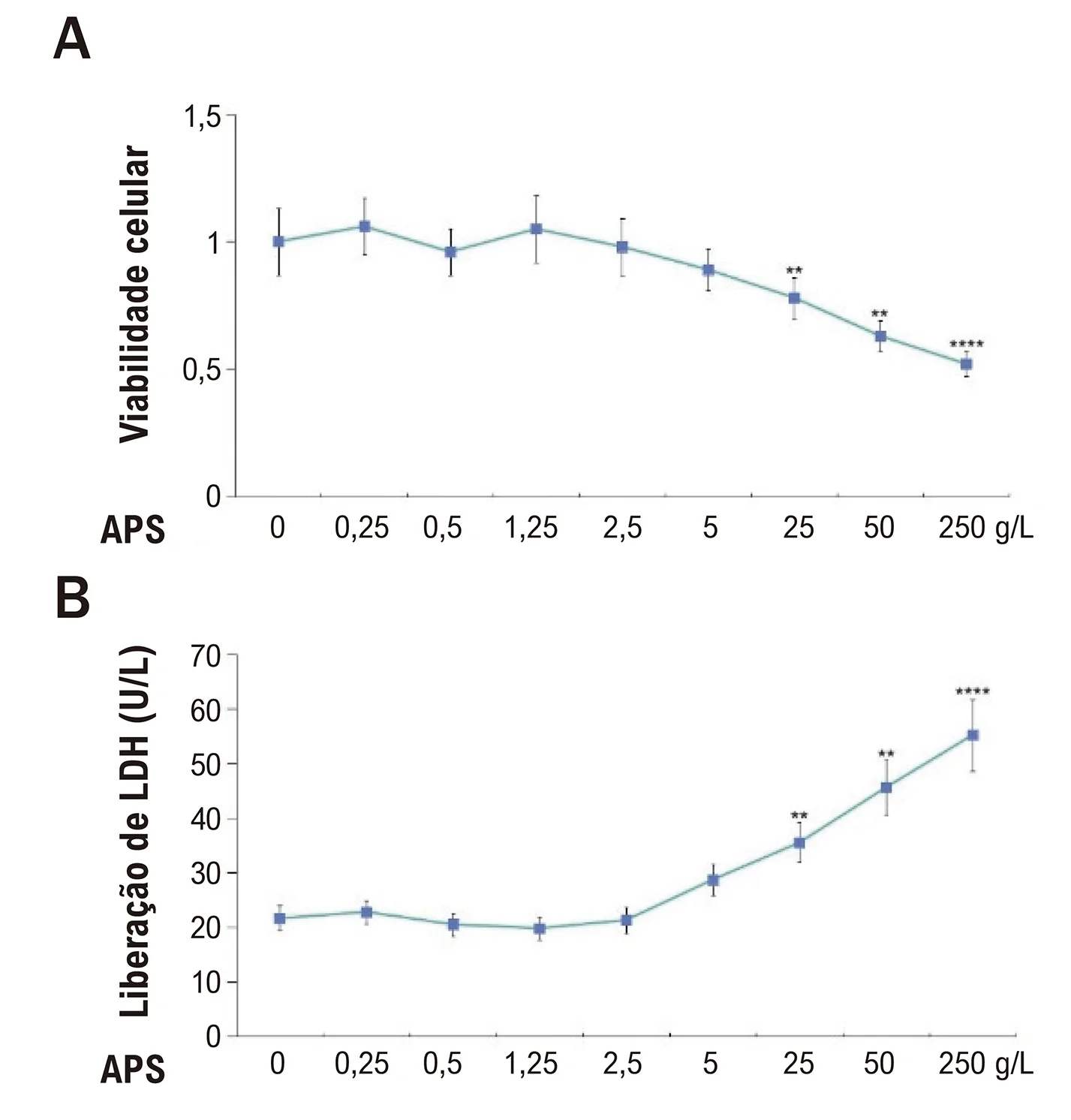
– Citotoxicidade de APS em Cardiomiócitos HL-1. As células foram estimuladas com APS nas concentrações de 0, 0,25, 0,5, 1,25, 2,5, 5, 25, 50 e 250 g/L durante 48 horas. A) A viabilidade celular foi medida usando o ensaio CCK-8 (n=6 por grupo). B) A liberação de LDH foi medida usando um kit comercial (n=6 por grupo) (**: p<0,01 vs. Grupo controle; ****: p<0,001 vs. Grupo controle).

### O APS melhora a função respiratória mitocondrial e a produção de ATP

Para determinar se o APS melhora a função mitocondrial, medimos a respiração mitocondrial e a produção de ATP. Células HL-1 foram tratadas com 1,25 ou 2,5 g/L de APS por 48 horas. O tratamento com APS aumentou significativamente a atividade do Complexo IV (citocromo c oxidase) de forma dose-dependente (Figura 2A). A análise com o software Seahorse XF revelou que as células tratadas com APS apresentaram respiração basal (Figura 2B) e respiração máxima (Figura 2C) significativamente maiores em comparação com os controles. Concordantemente, a produção intracelular de ATP aumentou acentuadamente após a exposição ao APS (Figura 2D). Esses resultados demonstram que o APS aumenta a fosforilação oxidativa mitocondrial e a produção bioenergética em cardiomiócitos.

**Figura 2:**
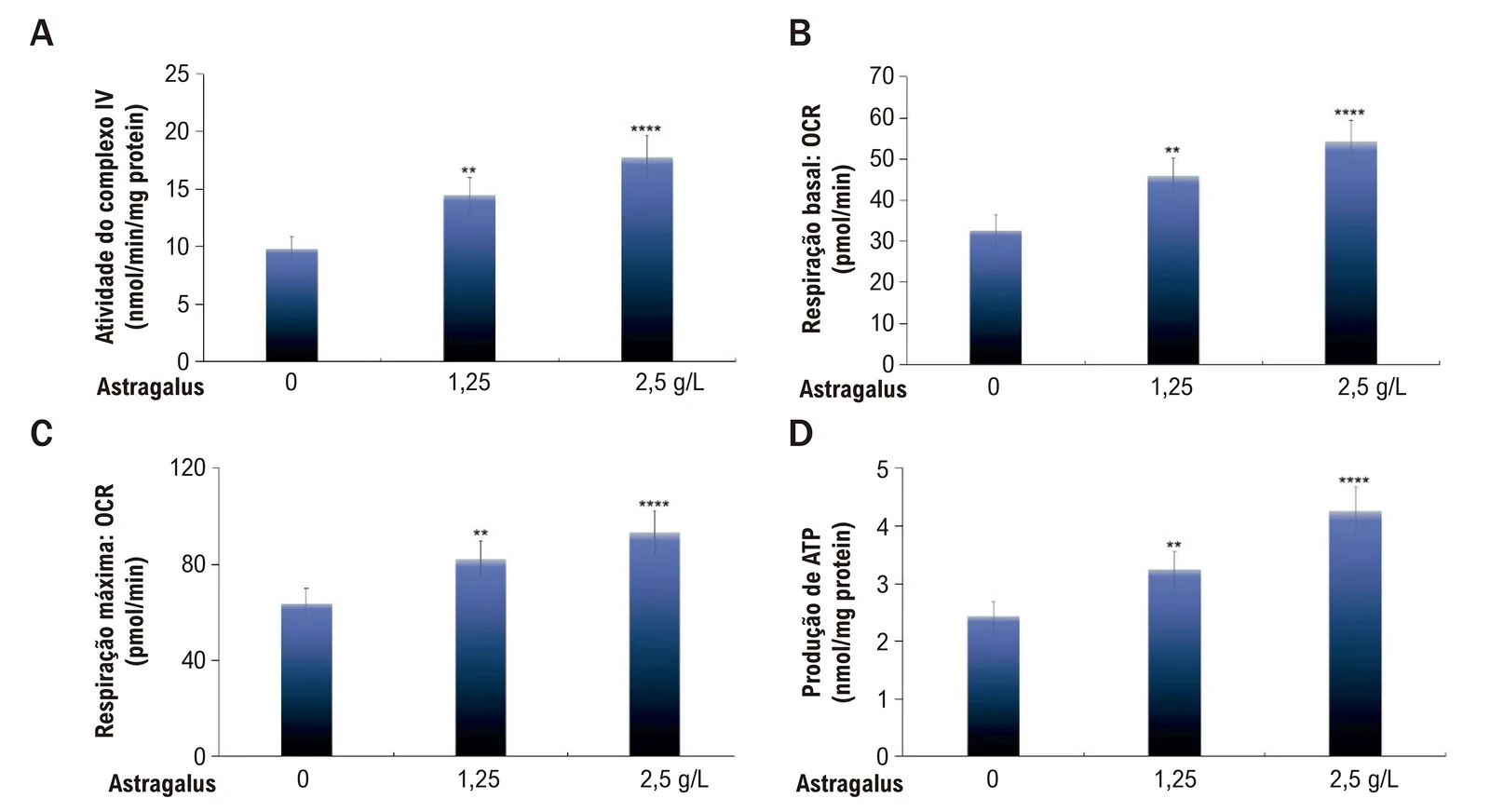
– O APS melhora a função mitocondrial em Cardiomiócitos HL-1. As células foram estimuladas com APS nas concentrações de 1,25 e 2,5 g/L durante 48 horas. A). Atividade do Complex IV (n=6 por grupo); B) Respiração basal: OCR (n=6 por grupo); C) Respiração máxima: OCR (n=6 por grupo); D) Produção de ATP (n=6 por grupo) (**: p<0,01 vs. Grupo controle; ****: p<0,001 vs. Grupo controle).

### APS promove a biogênese mitocondrial em cardiomiócitos

Em seguida, investigamos se o APS estimula a biogênese mitocondrial. A análise por qPCR revelou um aumento significativo na razão mtDNA/nDNA em células tratadas com APS (Figura 3A), indicando uma replicação mitocondrial aumentada. Paralelamente, a expressão da proteína citocromo B (Figura 3B) e a massa mitocondrial, avaliada pela coloração com MitoTracker Red (Figura 3C), estavam ambas elevadas no grupo APS. Esses resultados corroboram o papel do APS na promoção da biogênese mitocondrial em cardiomiócitos.

**Figura 3:**
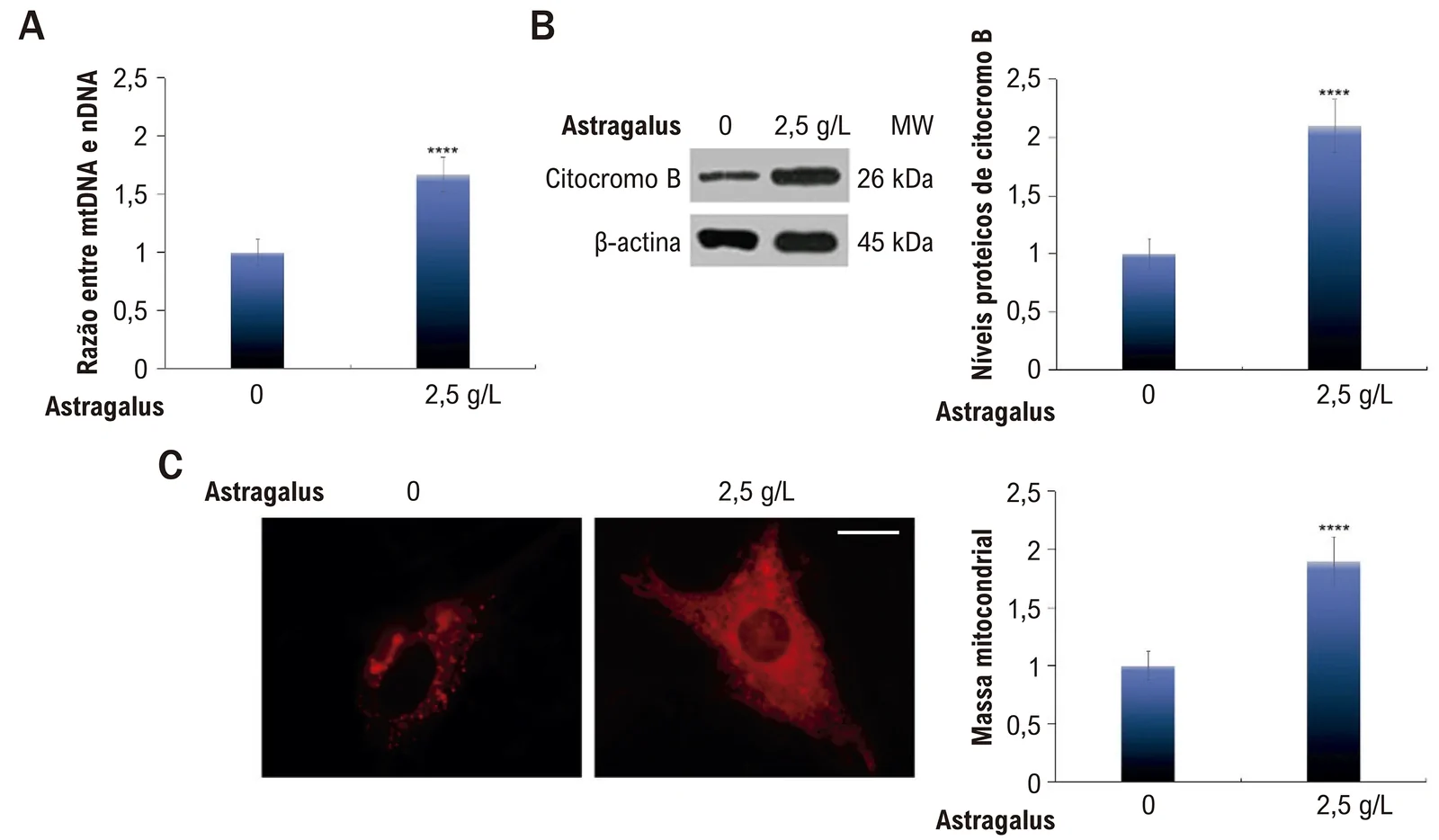
– APS promove a biogênese mitocondrial em Cardiomiócitos HL-1. As células foram estimuladas com APS a uma concentração de 2,5 g/L durante 48 horas. A) A razão entre o DNA mitocondrial (mtDNA) e o DNA nuclear (nDNA) (n=6 por grupo); B) Níveis de proteína do citocromo B (n=3 por grupo); C) Massa mitocondrial medida com coloração MitoTracker vermelho (Barras de escala = 10 μm, n=6 por grupo) (****: <0,001 vs. Grupo controle).

### APS regula positivamente genes e proteínas relacionados à biogênese mitocondrial

Para elucidar os mecanismos moleculares subjacentes à biogênese mitocondrial induzida por APS, avaliamos a expressão de reguladores canônicos. O tratamento com APS aumentou significativamente os níveis de mRNA (Figura 4A) e proteína (Figura 4B) do fator respiratório nuclear 2 (NRF2), do fator de transcrição mitocondrial A (TFAM) e da proteína desacopladora 2 (UCP2). Esses dados sugerem que o APS ativa a biogênese mitocondrial nos níveis transcricional e translacional.

**Figura 4:**
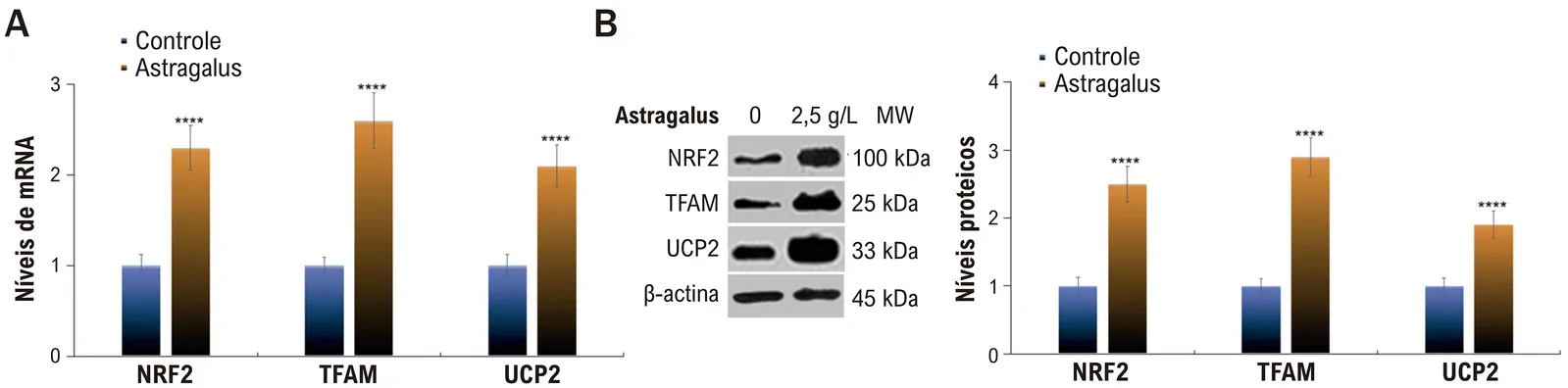
– O APS promove a expressão de reguladores da biogênese mitocondrial. As células foram estimuladas com APS a uma concentração de 2,5 g/L durante 48 horas. A) Níveis de mRNA de NRF2, TFAM e UCP2 (n=6 por grupo); B) Níveis de proteína de NRF2, TFAM e UCP2 (n=3 por grupo) (****, p<0,001 vs. Grupo de controle).

### APS induz a expressão de PGC-1α

Considerando o papel central do PGC-1α na regulação da biogênese mitocondrial, avaliamos sua expressão em resposta ao APS. Tanto os níveis de mRNA (Figura 5A) quanto os de proteína (Figura 5B) do PGC-1α foram significativamente aumentados de forma dose-dependente nas células tratadas com APS. Esses achados indicam que o PGC-1α é um mediador chave do aumento da atividade mitocondrial induzido pelo APS.

**Figura 5:**
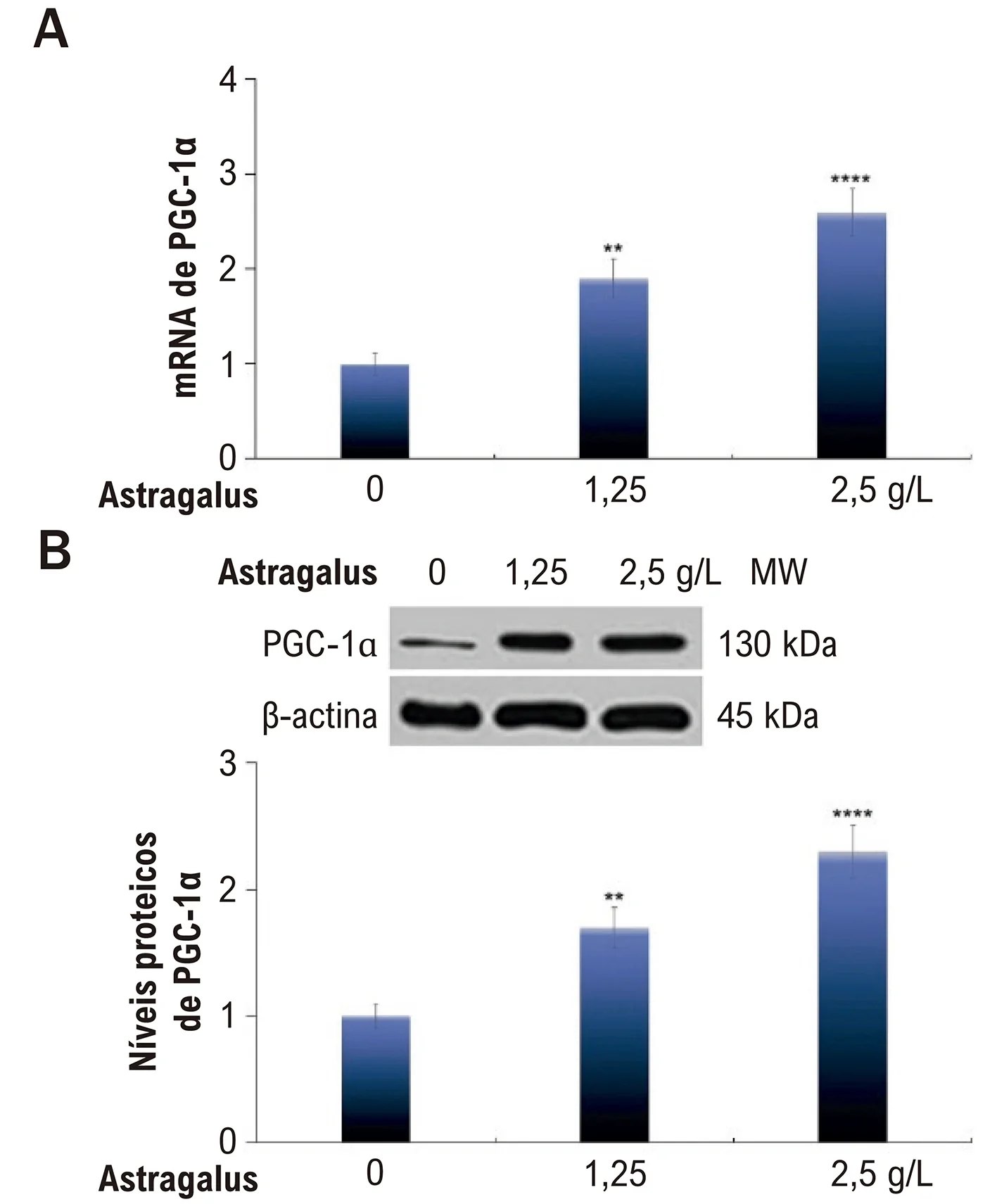
– APS aumenta a expressão de PGC-1α em Cardiomiócitos HL-1. As células foram estimuladas com APS nas concentrações de 1,25 e 2,5 g/L durante 48 horas. (A). mRNA de PGC-1ɑ (n=6 por grupo); (B) Níveis de proteína de PGC-1ɑ (n=3 por grupo) (**, p<0,01 vs. Grupo controle; ****, p<0,001 vs. Grupo controle).

### O APS ativa a via de sinalização AMPK em cardiomiócitos HL-1

Para determinar se a via AMPK está envolvida nos efeitos mediados por APS, examinamos o estado de fosforilação da AMPK e de seu efetor a jusante, a acetil-CoA carboxilase (ACC). A análise por Western blot mostrou que o tratamento com APS aumentou significativamente a fosforilação tanto da AMPKα quanto da ACC (Figura 6), indicando a ativação da cascata de sinalização da AMPK em cardiomiócitos.

**Figura 6:**
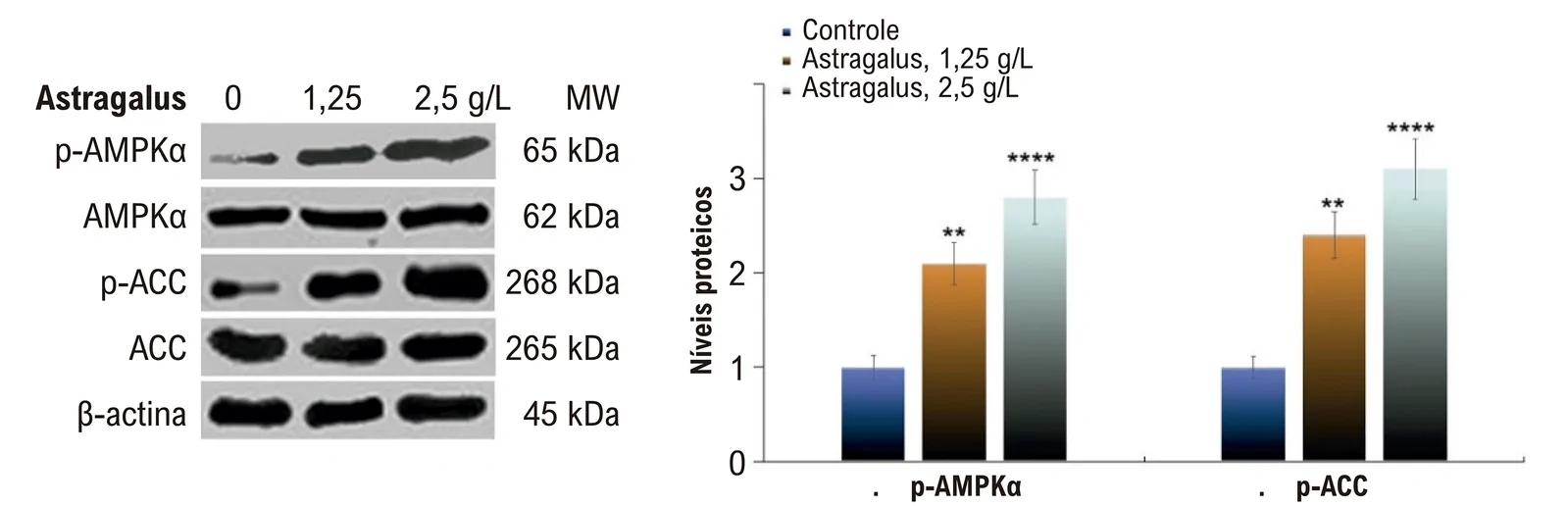
– APS promove a ativação da AMPK em Cardiomiócitos HL-1. As células foram estimuladas com APS nas concentrações de 1,25 e 2,5 g/L durante 48 horas. Os níveis de p-AMPKα e p-ACC foram medidos por análise de Western blot (n=3 por grupo) (**, p<0,01 vs. Grupo controle; ****, p<0,001 vs. Grupo controle).

### A inibição da AMPK elimina a proteção mitocondrial mediada por APS

Para confirmar o papel da AMPK nos efeitos mitocondriais mediados por APS, tratamos as células simultaneamente com APS (2,5 g/L) e o inibidor de AMPK, Composto C (10 μM). A inibição da AMPK suprimiu significativamente a superexpressão de mRNA de PGC-1α, NRF2, TFAM e UCP2 induzida por APS (Figuras 7A e 7B), e reduziu tanto a razão mtDNA/nDNA (Figura 7C) quanto a produção de ATP (Figura 7D). Esses resultados confirmam que a ativação da AMPK é essencial para os efeitos protetores mitocondriais do APS.

**Figura 7:**
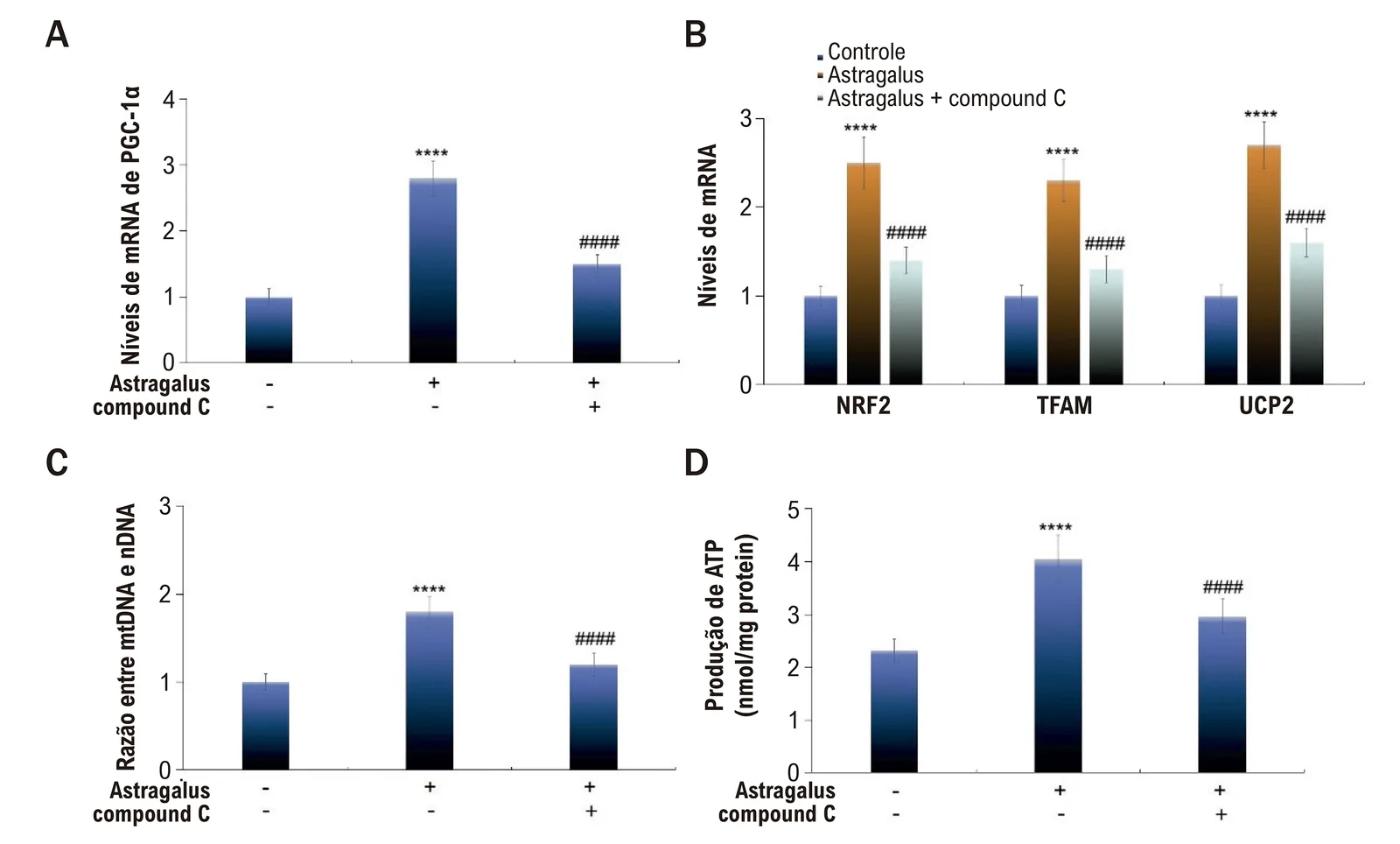
– A inibição da AMPK anulou os efeitos do APS na biogênese mitocondrial. As células foram estimuladas com 2,5 g/L de APS e o inibidor da AMPK, Composto C (10 μM), durante 48 horas. A) Níveis de mRNA de PGC-1α (n=6 por grupo); B) Níveis de mRNA de NRF2, TFAM e UCP2 (n=6 por grupo); C) Razão entre DNA mitocondrial (mtDNA) e DNA nuclear (nDNA) (n=6 por grupo); D) Produção de ATP (n=6 por grupo) (****, p<0,001 vs. Grupo controle; ####, p<0,001 vs. grupo APS).

## Discussão

Neste estudo, demonstramos que o APS, um composto bioativo natural extraído de *Astragalus membranaceus*, melhora significativamente a função mitocondrial e estimula a biogênese mitocondrial em cardiomiócitos HL-1. Mecanisticamente, esses efeitos são mediados pela ativação do eixo de sinalização da proteína quinase ativada por AMP (AMPK)/PGC-1α, destacando o potencial do APS como um agente terapêutico direcionado à mitocôndria para doenças cardiovasculares.

A disfunção mitocondrial desempenha um papel central na patogênese da insuficiência cardíaca, na qual a fosforilação oxidativa prejudicada leva à redução da produção de ATP, ao aumento da geração de ROS e à ativação de vias de morte celular, incluindo apoptose e necrose.^27,28^ Restaurar a homeostase mitocondrial por meio do aumento da biogênese e da reprogramação bioenergética emergiu como uma estratégia promissora para melhorar o desempenho cardíaco. Aqui, mostramos que o APS, em baixas concentrações (1,25–2,5 g/L), apresenta efeitos protetores contra lesões citotóxicas. Esses achados são consistentes com estudos anteriores que demonstraram as propriedades citoprotetoras e antiapoptóticas do APS em cardiomiócitos. Por exemplo, Sun et al. relataram que o APS atenuou a lesão induzida por estresse oxidativo em cardiomiócitos H9c2, suprimindo a NADPH oxidase e aumentando a atividade de enzimas antioxidantes.^29^ De forma semelhante, Cao et al. demonstraram que o APS protege contra a cardiotoxicidade induzida pela doxorrubicina, reduzindo a apoptose e a disfunção mitocondrial por meio da ativação da via AMPK/mTOR.^30^ Esses relatórios, em conjunto, corroboram nossas observações de que o APS exerce efeitos protetores diretos sobre os cardiomiócitos em condições de estresse celular.

É importante destacar que o APS melhora significativamente a função mitocondrial. Nossos resultados mostram que o APS aumenta a atividade do Complexo IV, melhora a respiração basal e máxima e eleva os níveis intracelulares de ATP. Esses resultados refletem uma maior eficiência respiratória mitocondrial. Melhorias semelhantes na respiração mitocondrial foram relatadas em resposta a outros compostos de origem vegetal, como a berberina e o resveratrol, que também atuam na bioenergética mitocondrial por meio da sinalização da AMPK.

Também foi constatado que o APS promove a biogênese mitocondrial, como evidenciado pelo aumento no número de cópias de mtDNA, na expressão da proteína citocromo B (Figura 3B) e na massa mitocondrial. Essas alterações fenotípicas são acompanhadas pela regulação positiva de reguladores mestres da biogênese mitocondrial, incluindo PGC-1α, NRF2, TFAM e UCP2. Notavelmente, o PGC-1α orquestra programas transcricionais que impulsionam a replicação do mtDNA e a capacidade oxidativa, e sua elevação representa uma assinatura molecular chave da biogênese.^10,31-33^ Nossos resultados estão de acordo com relatos que mostram que a ativação farmacológica do PGC-1α aumenta a renovação mitocondrial e melhora a flexibilidade metabólica cardíaca.

Mecanisticamente, o APS ativou a AMPK e seu alvo a jusante, a ACC, confirmando seu papel como modulador sensor de energia. A necessidade da ativação da AMPK foi validada utilizando o Composto C, que aboliu a regulação positiva induzida por APS da PGC-1α, de genes relacionados à biogênese mitocondrial e da síntese de ATP. A AMPK é bem estabelecida como um regulador central da biogênese mitocondrial e do equilíbrio energético celular, e sua ativação por produtos naturais já foi observada no contexto da síndrome metabólica e da cardiopatia isquêmica. No entanto, permanece incerto se o APS interage diretamente com a AMPK ou age indiretamente por meio da modulação de quinases a montante, como LKB1 ou CaMKKβ, ou por meio da sinalização dependente de ROS/Ca^2^⁺.^34,35^

Nossos resultados ampliam trabalhos anteriores, que se concentraram principalmente nos efeitos anti-inflamatórios ou antioxidantes do APS, fornecendo evidências mecanísticas diretas que ligam o APS à remodelação mitocondrial. Curiosamente, o APS demonstrou inibir lesões induzidas por estresse oxidativo na cardiomiopatia diabética por meio da supressão da NADPH oxidase e da sinalização do TGF-β, sugerindo que as vias mitocondriais podem atuar em paralelo com as vias canônicas de estresse e fibrose.^29^

No entanto, algumas limitações devem ser consideradas. Primeiro, todos os experimentos foram conduzidos in vitro utilizando a linhagem celular de cardiomiócitos murinos HL-1. Embora as células HL-1 retenham muitas características dos cardiomiócitos primários e sejam amplamente utilizadas em pesquisas cardiovasculares, elas são uma linhagem celular imortalizada e podem não replicar completamente o complexo ambiente metabólico, biomecânico e neuro-humoral do miocárdio in vivo. Portanto, a validação de nossos achados em cardiomiócitos primários e, mais importante, em modelos animais apropriados de insuficiência cardíaca ou disfunção mitocondrial é essencial antes de se chegar a conclusões definitivas sobre o potencial terapêutico do APS. Segundo, embora tenhamos identificado a AMPK como um mediador chave, o mecanismo inicial de detecção pelo qual o APS desencadeia a ativação da AMPK ainda precisa ser determinado. Finalmente, a farmacocinética, o direcionamento tecidual e a segurança a longo prazo do APS não foram adequadamente avaliados. Dada a sua natureza polissacarídica, a eficiência de captação celular e a estabilidade metabólica in vivo requerem investigação adicional.

## Conclusão

Em conclusão, nossos resultados revelam que o APS promove a biogênese mitocondrial e melhora a função respiratória por meio da ativação da via de sinalização AMPK/PGC-1α. Este estudo fornece novos insights mecanísticos sobre os efeitos protetores mitocondriais do APS e estabelece as bases para futuros estudos translacionais em distúrbios metabólicos cardíacos. A validação desses achados em modelos pré-clínicos de insuficiência cardíaca e a elucidação da sinalização a montante serão cruciais para estabelecer o APS como uma estratégia terapêutica viável.

## Material suplementar

Material suplementar Para informação adicional, por favor, clique aqui.
