# Supplementary material for: O Polissacarídeo de Astragalus Melhora a Biogênese e a Função Mitocondrial em Cardiomiócitos através da Ativação da AMPK
Source: Arq Bras Cardiol. 2026 Sep 18;123(8):e20250854. [Article in Portuguese] doi: 10.36660/abc.20250854 (PMC13671154; doi:10.36660/abc.20250854)
Supplement: Supplementary file 1 [file 0066-782x-abc-123-8-e20250854-suppl01.pdf]

Supplementary Materials

Table S1. Primer sequences used for quantitative real-time PCR

Gene	Forward primer (5'-3')	Reverse primer (5'-3')
PGC-1α	TATGGAGTGACATAGAGTGTGCT	GGTTATCTTCGTAGGTGTGAGG
NRF2	CGCAGACATTCCCATTTGTAG	TCTTGCTCTTGTTTTCGGTTC
TFAM	AGAGATCAGGGAGGACAAAGTG	GTGTGAGGAGGATGGCTTTGA
UCP2	AGGGCTCTACGACTCCTTAC	TGCAGTGGAGGAGGTTGTA
Cytochrome B	TTATTGTGGGAGTAGATTGGGTC	GAGGGGTAGGAGAGGTGATTG
β-actin	CATTGCTGACAGGATGCAGAAG	TGCTGGAAGGTGGACAGTGAG
GAPDH	AGGTCGGTGTGAACGGATTTG	TGTAGACCATGTAGTTGAGGTCA

Table S2. Antibodies used for Western blot analysis

Target protein	Source	Catalog number	Dilution
PGC-1α	Cell Signaling Technology	#2178	1:1000
NRF2	Cell Signaling Technology	#12721	1:1000
TFAM	Abcam	ab131607	1:1000
UCP2	Abcam	ab67241	1:1000
p-AMPKα (Thr172)	Cell Signaling Technology	#2535	1:1000
p-ACC (Ser79)	Cell Signaling Technology	#11818	1:1000
β-actin	Cell Signaling Technology	#4967	1:5000
HRP-conjugated secondary antibodies	Cell Signaling Technology	#7074 (anti-rabbit), #7076 (anti-mouse)	1:5000
