# Supplementary material for: O Polissacarídeo de Astragalus Melhora a Biogênese e a Função Mitocondrial em Cardiomiócitos através da Ativação da AMPK
Source: Arq Bras Cardiol. 2026 Sep 18;123(8):e20250854. [Article in Portuguese] doi: 10.36660/abc.20250854 (PMC13671154; doi:10.36660/abc.20250854)
Supplement: Translation

# Translation: Astragalus Polysaccharide Enhances Mitochondrial Biogenesis and Function in Cardiomyocytes Through AMPK Activation

**DOI:** 10.36660/abc.20250854i

**Authors:** Chengbin Wang, Shuhan Lv, Xingde Liu

**Affiliations:** 1 China Guizhou University of Traditional Chinese Medicine – Department of Second Clinical Medical College, Guiyang – China

**Keywords:** Cardiac Myocytes, Organelle Biogenesis, Heart Failure

## Abstract

**Background:**

Mitochondrial dysfunction is a key driver of heart failure, underscoring the need for therapies that promote mitochondrial biogenesis and function. Astragalus polysaccharide (APS), a bioactive compound from Astragalus membranaceus, has demonstrated cardioprotective properties, but its direct effects on mitochondrial homeostasis in cardiomyocytes remain unclear.

**Objectives:**

This study aimed to investigate the effects of APS on mitochondrial function and biogenesis in HL-1 cardiomyocytes and to elucidate the involvement of the AMP-activated protein kinase (AMPK)/peroxisome proliferator-activated receptor gamma coactivator 1-alpha (PGC-1α) signaling pathway.

**Methods:**

HL-1 cardiomyocytes were treated with non-toxic concentrations of APS (1.25-2.5 g/L) for 48 hours. Mitochondrial respiration was assessed via Seahorse XF Analyzer, adenosine triphosphate (ATP) levels were quantified, and Complex IV activity was measured. Mitochondrial biogenesis was evaluated by mitochondrial DNA (mtDNA)/ nuclear DNA (nDNA) ratio, cytochrome B levels, and mitochondrial mass. Key proteins and genes, including PGC-1α, nuclear respiratory factor 2 (NRF2), mitochondrial transcription factor A (TFAM), uncoupling protein 2 (UCP2), and phosphorylated AMPK (p-AMPK), were analyzed by Western blot and qPCR. The AMPK inhibitor Compound C was used to confirm the pathway’s role.

**Results:**

APS treatment significantly enhanced mitochondrial respiration, ATP production, and Complex IV activity. It promoted mitochondrial biogenesis, evidenced by increased mtDNA/nDNA ratio and mitochondrial mass. APS upregulated the expression of PGC-1α, NRF2, TFAM, and UCP2, and activated AMPK phosphorylation. All these beneficial effects were abolished by AMPK inhibition with Compound C.

**Conclusions:**

APS enhances mitochondrial biogenesis and function in cardiomyocytes through activation of the AMPK/PGC-1α signaling pathway, identifying it as a potential therapeutic agent for cardiac diseases associated with mitochondrial dysfunction.

## Introduction

Mitochondrial dysfunction is a hallmark of numerous cardiovascular diseases, including heart failure, ischemic cardiomyopathy, and diabetic cardiomyopathy. In cardiomyocytes, which possess one of the highest energy demands among cell types, mitochondrial integrity is essential for maintaining contractile function, calcium homeostasis, and redox balance.^1-3^ Impairment in mitochondrial oxidative phosphorylation leads to adenosine triphosphate (ATP) depletion, excessive reactive oxygen species (ROS) production, and eventual cardiomyocyte death, thereby contributing to the progression of heart failure.^4,5^ To compensate for mitochondrial damage or loss, cells activate mitochondrial biogenesis—a tightly regulated process involving the replication of mitochondrial DNA (mtDNA), expansion of mitochondrial mass, and upregulation of nuclear-encoded mitochondrial proteins.^6-9^ Central to this process is peroxisome proliferator-activated receptor gamma coactivator 1-alpha (PGC-1α), a transcriptional coactivator that orchestrates mitochondrial gene expression by interacting with nuclear respiratory factors (NRFs) and mitochondrial transcription factor A (TFAM).^10,11^ However, in pathological conditions, this adaptive mechanism is often blunted, leading to persistent mitochondrial insufficiency. Therefore, pharmacological strategies that restore or enhance PGC-1α-driven mitochondrial biogenesis have emerged as promising therapeutic approaches for mitochondrial cardiomyopathies.

Astragalus polysaccharide (APS), a major bioactive component of the traditional Chinese medicinal herb *Astragalus membranaceus*, has attracted considerable attention for its cardioprotective, antioxidant, and anti-inflammatory properties.^12-15^ APS has been reported to ameliorate myocardial injury in models of ischemia–reperfusion, diabetic cardiomyopathy, and hypertensive heart disease. Several studies have shown that APS modulates cellular signaling pathways, including phosphoinositide 3-kinase (PI3K)/protein kinase B (Akt), nuclear factor kappa-B (NF-κB), and transforming growth factor-beta 1 (TGF-β1), suggesting its pleiotropic regulatory potential.^16-19^ Accumulating evidence highlights the potential of natural products, including flavonoids, saponins, and polysaccharides, to modulate mitochondrial function and biogenesis in cardiovascular diseases. Recent reviews have emphasized that natural compounds can target mitochondrial dynamics, reduce oxidative stress, and regulate mitophagy, thereby exerting cardioprotective effects.^20,21^ In particular, plant-derived compounds such as berberine, resveratrol, and various herbal extracts have been shown to activate the AMP-activated protein kinase (AMPK)/PGC-1α signaling pathway, promoting mitochondrial biogenesis and improving cardiac function.^22^ However, its precise role in mitochondrial biology, particularly in relation to mitochondrial biogenesis and energy metabolism in cardiomyocytes, remains largely unexplored. Given the critical role of mitochondrial homeostasis in cardiac function, we hypothesized that APS promotes mitochondrial biogenesis and function in cardiomyocytes through activation of the AMPK/PGC-1α signaling pathway. To test this hypothesis, we investigated the effects of APS on mitochondrial function and biogenesis in HL-1 cardiomyocytes and elucidated the underlying molecular mechanisms (Central Illustration).

## Materials and Methods

### Cell culture and treatment

HL-1 murine cardiomyocytes (Millipore, USA) were cultured in Claycomb medium supplemented with 10% fetal bovine serum (FBS), 100 U/mL penicillin, 100 μg/mL streptomycin, and 0.1 mM norepinephrine (Sigma-Aldrich, USA). Cells were maintained in a humidified incubator at 37 °C with 5% CO_2_. For treatment, APS (purity ≥98%, Yuanye Bio-Technology, China) was dissolved in sterile water to prepare a stock solution of 100 g/L, which was then filtered through a 0.22 μm sterile filter and diluted to the indicated concentrations (0–250 g/L) with culture medium immediately before use. Cells were treated for 48 hours. In selected experiments, Compound C (MedChemExpress, USA) was dissolved in dimethyl sulfoxide (DMSO) to prepare a 10 mM stock solution and stored at −20°C. Immediately before use, the stock solution was diluted in culture medium to a final concentration of 10 μM (with a final DMSO concentration <0.1%). Cells were pretreated with Compound C for 1 hour before APS administration.

### CCK-8 assay

Cell viability was assessed using the Cell Counting Kit-8 (Dojindo, Japan) according to the manufacturer’s instructions. HL-1 cells were seeded in 96-well plates (1 × 10^4^ cells/well) and treated with APS at concentrations of 0, 0.25, 0.5, 1.25, 2.5, 5, 25, 50, and 250 g/L for 48 hours. After treatment, 10 μL of CCK-8 reagent was added to each well and incubated for 2 hours at 37°C in the dark. Absorbance was measured at 450 nm using a microplate reader (BioTek, USA).

### LDH release

Lactate dehydrogenase (LDH) activity in the culture supernatant was measured using a commercial LDH Cytotoxicity Assay Kit (Beyotime, China) according to the manufacturer’s instructions, as previously described for cytotoxicity assessment.^23^ Following treatment, 100 μL of culture medium was collected from each well, and LDH activity was determined spectrophotometrically at 490 nm. Results were expressed as U/L.

### Complex IV activity

Cytochrome c oxidase (Complex IV) activity was measured using a colorimetric enzyme activity assay kit (Solarbio, China) following the manufacturer’s protocol, as previously described.^24^ HL-1 cells were lysed, and the enzymatic activity was quantified at 550 nm. Data were normalized to protein content and expressed as nmol/min/mg protein.

### Measurement of mitochondrial respiratory function

Mitochondrial respiration was evaluated using a Seahorse XFe24 Analyzer (Agilent Technologies, USA). Basal and maximal oxygen consumption rates (OCR) were measured using the Seahorse XF Cell Mito Stress Test Kit, as previously described, with sequential injection of oligomycin (1 μM), carbonyl cyanide-4-(trifluoromethoxy) phenylhydrazone (FCCP, 1 μM), and rotenone/antimycin A (0.5 μM each).^25^ All compounds were dissolved according to the manufacturer’s instructions: oligomycin and FCCP were dissolved in DMSO to prepare 10 mM stock solutions, while rotenone and antimycin A were dissolved in DMSO to prepare 5 mM stock solutions. Stock solutions were aliquoted and stored at −20°C, then diluted to working concentrations in Seahorse assay medium immediately before use. HL-1 cells were seeded in Seahorse plates and treated with APS (1.25 or 2.5 g/L) for 48 hours before measurement. OCR values were normalized to protein concentration.

### Determination of ATP production

Intracellular ATP levels were quantified using an ATP assay kit (Beyotime, China) based on a luciferase-catalyzed bioluminescence reaction, as described previously.^26^ Cells were lysed in ATP lysis buffer, and luminescence intensity was measured using a luminometer (Promega, USA). Protein concentrations were determined using the bicinchoninic acid (BCA) assay for normalization. ATP content was normalized to total protein and expressed as nmol/mg protein.

### Quantification of the mtDNA to nDNA ratio

Total DNA was extracted using the TIANamp Genomic DNA Kit (Tiangen, China). The relative mtDNA content was quantified by real-time PCR using primers targeting the mitochondrial cytochrome B gene and the nuclear β-actin gene. The mtDNA/nDNA ratio was calculated using the 2^⁻Ct^method.

### MitoTracker Red staining

Mitochondrial mass was assessed using MitoTracker Red CMXRos (Thermo Fisher Scientific, USA). The MitoTracker Red dye was dissolved in high-quality anhydrous DMSO to prepare a 1 mM stock solution, aliquoted, and stored at −20°C protected from light. Immediately before use, the stock solution was diluted in pre-warmed serum-free culture medium to a final concentration of 100 nM. Cells were incubated with the staining solution for 30 minutes at 37°C in the dark, washed twice with PBS, and visualized using a fluorescence microscope (Olympus IX73, Japan). For quantitative analysis, fluorescence intensity was measured by flow cytometry (BD FACSCanto II).

### Real-time PCR (qPCR)

Total RNA was extracted using TRIzol reagent (Invitrogen, USA), and cDNA synthesis was performed using a PrimeScript RT reagent kit (Takara, Japan). qPCR was carried out with SYBR Green Master Mix (Roche, Switzerland) on a LightCycler 480 system. The thermal cycling conditions were as follows: initial pre-incubation at 95°C for 10 minutes, followed by 40 cycles of denaturation at 95°C for 15 seconds, annealing at 60°C for 30 seconds, and extension at 72°C for 30 seconds. A melt curve analysis was performed at the end of each run to verify amplification specificity. Gene expression levels of PGC-1α, NRF2, TFAM, UCP2, and cytochrome B were normalized to glyceraldehyde-3-phosphate dehydrogenase (GAPDH) using the 2^⁻Ct^ method. Primer sequences are listed in Supplementary Table S1.

### Western blot analysis

Total protein was extracted with radioimmunoprecipitation assay (RIPA) lysis buffer containing protease and phosphatase inhibitors (Beyotime, China). Equal amounts of protein (30 μg) were separated by sodium dodecyl sulfate–polyacrylamide gel electrophoresis (SDS-PAGE) using 12% gels, and transferred onto polyvinylidene fluoride (PVDF) membranes (Millipore, USA). Membranes were blocked in 5% non-fat milk for 1 hour at room temperature and then incubated overnight at 4 °C with primary antibodies against PGC-1α, NRF2, TFAM, UCP2, phospho-AMPKα (Thr172), phospho-ACC (Ser79), and β-actin (all from Cell Signaling Technology, USA). HRP-conjugated secondary antibodies were used, and bands were visualized using enhanced chemiluminescence (ECL, Thermo Fisher) with exposure times of 30 seconds to 2 minutes, depending on signal intensity. Protein bands were quantified by densitometry using ImageJ software (National Institutes of Health, USA), with signal intensities normalized to β-actin loading controls. Results were expressed as fold change relative to the control group. Antibody information is listed in Supplementary Table S2.

### Statistical analysis

All data are expressed as mean ± standard deviation (SD) from at least three independent experiments. The data distribution normality was tested using the Shapiro-Wilk test. For experiments involving a single factor, statistical significance was assessed using one-way analysis of variance (ANOVA) followed by Tukey’s post hoc test for multiple comparisons. For experiments involving two factors, two-way ANOVA was performed to evaluate main effects and interactions, followed by Tukey’s multiple comparisons test. A p-value < 0.05 was considered statistically significant. Analyses were performed using GraphPad Prism 9.0 (GraphPad Software, USA).

## Results

### APS exhibits low cytotoxicity at low concentrations

Before evaluating the effects of APS on mitochondrial function, we first assessed its cytotoxicity in HL-1 cardiomyocytes. Cells were exposed to increasing concentrations of APS (0–250 g/L) for 48 hours. As shown by the CCK-8 assay, low concentrations of APS (1.25–2.5 g/L) did not significantly affect cell viability compared to the control (Figure 1A), whereas high concentrations (≥25 g/L) led to a significant, dose-dependent decrease in viability. Consistently, LDH release assays revealed minimal cytotoxicity at 1.25 and 2.5 g/L, but LDH activity markedly increased at higher concentrations (Figure 1B). These data suggest that APS is well tolerated at low concentrations and may exert cytoprotective effects under non-toxic conditions.

**Figure 1:** – Cytotoxicity of APS in HL-1 Cardiomyocytes. Cells were stimulated with APS at the concentrations of 0, 0.25, 0.5, 1.25, 2.5, 5, 25, 50, 250 g/L for 48 hours. (A). Cell viability was measured using CCK-8 assay (n=6 per group). (B). LDH release was measured using a commercial kit (n=6 per group) (**, P<0.01 vs. Control group; ****, P<0.001 vs. Control group).

### APS enhances mitochondrial respiratory function and ATP production

To determine whether APS improves mitochondrial function, we measured mitochondrial respiration and ATP production. HL-1 cells were treated with 1.25 or 2.5 g/L APS for 48 hours. APS treatment significantly increased Complex IV (cytochrome c oxidase) activity in a dose-dependent manner (Figure 2A). Seahorse XF analysis revealed that APS-treated cells exhibited significantly higher basal respiration (Figure 2B) and maximal respiration (Figure 2C) compared to controls. Concordantly, intracellular ATP production was markedly elevated following APS exposure (Figure 2D). These results demonstrate that APS enhances mitochondrial oxidative phosphorylation and bioenergetic output in cardiomyocytes.

**Figure 2:** – APS enhances mitochondrial function in HL-1 Cardiomyocytes. Cells were stimulated with APS at the concentrations of 1.25, 2.5 g/L for 48 hours. (A). Complex IV activity (n=6 per group); (B). Basal respiration: OCR (n=6 per group); (C). Maximal respiration: OCR (n=6 per group); (D). ATP production (n=6 per group) (**, P<0.01 vs. Control group; ****, P<0.001 vs. Control group).

### APS promotes mitochondrial biogenesis in cardiomyocytes

We next investigated whether APS stimulates mitochondrial biogenesis. qPCR analysis revealed a significant increase in the mtDNA to nDNA ratio in APS-treated cells (Figure 3A), indicative of enhanced mitochondrial replication. In parallel, cytochrome B protein expression (Figure 3B) and mitochondrial mass, as assessed by MitoTracker Red staining (Figure 3C), were both elevated in the APS group. These results support a role for APS in promoting mitochondrial biogenesis in cardiomyocytes.

**Figure 3:** – APS promotes mitochondrial biogenesis in HL-1 Cardiomyocytes. Cells were stimulated with APS at a concentration of 2.5 g/L for 48 hours. (A). The ratio of mitochondrial DNA (mtDNA) to nuclear DNA (nDNA) (n=6 per group); (B). Protein levels of cytochrome B (n=3 per group); (C). Mitochondrial mass measured with MitoTracker red staining (scale bars = 10 μm, n=6 per group) (****, P<0.001 vs. Control group).

### APS upregulates mitochondrial biogenesis-related genes and proteins

To elucidate the molecular mechanisms underlying APS-induced mitochondrial biogenesis, we assessed the expression of canonical regulators. APS treatment significantly increased both mRNA (Figure 4A) and protein levels (Figure 4B) of nuclear respiratory factor 2 (NRF2), mitochondrial transcription factor A (TFAM), and uncoupling protein 2 (UCP2). These data suggest that APS activates mitochondrial biogenesis at the transcriptional and translational levels.

**Figure 4:** – APS promotes the expression of mitochondrial biogenesis regulators. Cells were stimulated with APS at a concentration of 2.5 g/L for 48 hours. (A). mRNA levels of NRF2, TFAM, and UCP2 (n=6 per group); (B). Protein levels of NRF2, TFAM, and UCP2 (n=3 per group) (****, P<0.001 vs. Control group*).*

### APS induces PGC-1α expression

Given the central role of PGC-1α in regulating mitochondrial biogenesis, we evaluated its expression in response to APS. Both PGC-1α mRNA (Figure 5A) and protein levels (Figure 5B) were significantly and dose-dependently upregulated in APS-treated cells. These findings indicate that PGC-1α is a key mediator of APS-induced mitochondrial enhancement.

**Figure 5:** – APS increases the expression of PGC-1ɑ in HL-1 Cardiomyocytes. Cells were stimulated with APS at the concentrations of 1.25 and 2.5 g/L for 48 hours. (A). mRNA of PGC-1ɑ (n=6 per group); (B). Protein levels of PGC-1ɑ (n=3 per group) (**, P<0.01 vs. Control group; ****, P<0.001 vs. Control group).

### APS activates the AMPK signaling pathway in HL-1 cardiomyocytes

To determine whether the AMPK pathway is involved in APS-mediated effects, we examined the phosphorylation status of AMPK and its downstream effector acetyl-CoA carboxylase (ACC). Western blot analysis showed that APS treatment significantly increased the phosphorylation of both AMPKα and ACC (Figure 6), indicating activation of the AMPK signaling cascade in cardiomyocytes.

**Figure 6:** – APS promotes the activation of AMPK in HL-1 Cardiomyocytes. Cells were stimulated with APS at the concentrations of 1.25 and 2.5 g/L for 48 hours. The levels of p-AMPKɑ and p-ACC were measured by western blot analysis (n=3 per group) (**, P<0.01 vs. Control group; ****, P<0.001 vs. Control group).

### Inhibiting AMPK abolishes APS-mediated mitochondrial protection

To confirm the role of AMPK in APS-mediated mitochondrial effects, we co-treated cells with APS (2.5 g/L) and the AMPK inhibitor Compound C (10 μM). AMPK inhibition significantly suppressed APS-induced upregulation of PGC-1α, NRF2, TFAM, and UCP2 mRNA (Figure 7A and 7B), and reduced both mtDNA/nDNA ratio (Figure 7C) and ATP production (Figure 7D). These results confirm that AMPK activation is essential for the mitochondrial protective effects of APS.

**Figure 7:** – Inhibition of AMPK abolished the effects of APS in mitochondrial biogenesis. Cells were stimulated with 2.5 g/L APS and the AMPK inhibitor Compound C (10 μM) for 48 hours. (A). mRNA levels of PGC-1ɑ (n=6 per group); (B). mRNA levels of NRF2, TFAM and UCP2 (n=6 per group); (C). The ratio of mitochondrial DNA (mtDNA) to nuclear DNA (nDNA) (n=6 per group); (D). ATP production (n=6 per group) (****, P<0.001 vs. Control group; ####, P<0.001 vs. APS group).

## Discussion

In this study, we demonstrate that APS, a natural bioactive compound extracted from *Astragalus membranaceus*, markedly improves mitochondrial function and stimulates mitochondrial biogenesis in HL-1 cardiomyocytes. Mechanistically, these effects are mediated via activation of the AMP-activated protein kinase (AMPK)/PGC-1α signaling axis, highlighting the potential of APS as a mitochondria-targeted therapeutic agent for cardiovascular diseases.

Mitochondrial dysfunction plays a central role in the pathogenesis of heart failure, in which impaired oxidative phosphorylation leads to reduced ATP production, increased ROS generation, and activation of cell death pathways, including apoptosis and necrosis.^27,28^ Restoring mitochondrial homeostasis via enhanced biogenesis and bioenergetic reprogramming has emerged as a promising strategy to improve cardiac performance. Here, we show that APS, at low concentrations (1.25–2.5 g/L), displays protective effects against cytotoxic injury. These findings are consistent with previous studies demonstrating the cytoprotective and anti-apoptotic properties of APS in cardiomyocytes. For instance, Sun et al. reported that APS attenuated oxidative stress-induced injury in H9c2 cardiomyocytes by suppressing NADPH oxidase and enhancing antioxidant enzyme activity.^29^ Similarly, Cao et al. showed that APS protected against doxorubicin-induced cardiotoxicity by reducing apoptosis and mitochondrial dysfunction via activation of the AMPK/mTOR pathway.^30^ These reports collectively support our observations that APS exerts direct protective effects on cardiomyocytes under conditions of cellular stress.

Importantly, APS significantly enhances mitochondrial function. Our results show that APS increases Complex IV activity, enhances basal and maximal respiration, and upregulates intracellular ATP levels. These outcomes reflect improved mitochondrial respiratory efficiency. Similar enhancements of mitochondrial respiration have been reported in response to other herbal compounds such as berberine and resveratrol, which also target mitochondrial energetics via AMPK signaling.

APS was also found to promote mitochondrial biogenesis, as evidenced by increases in mtDNA copy number, cytochrome B protein expression (Figure 3B), and mitochondrial mass. These phenotypic alterations are paralleled by the upregulation of master regulators of mitochondrial biogenesis, including PGC-1α, NRF2, TFAM, and UCP2. Notably, PGC-1α orchestrates transcriptional programs that drive mtDNA replication and oxidative capacity, and its elevation represents a key molecular signature of biogenesis.^10,31-33^ Our findings align with reports showing that pharmacological activation of PGC-1α enhances mitochondrial turnover and improves cardiac metabolic flexibility.

Mechanistically, APS activated AMPK and its downstream target ACC, confirming its role as an energy-sensing modulator. The necessity of AMPK activation was validated using Compound C, which abolished APS-induced upregulation of PGC-1α, mitochondrial biogenesis-related genes, and ATP synthesis. AMPK is well established as a central regulator of mitochondrial biogenesis and cellular energy balance, and its activation by natural products has been previously observed in the context of metabolic syndrome and ischemic heart disease. However, whether APS directly interacts with AMPK or acts indirectly through modulation of upstream kinases such as LKB1 or CaMKKβ, or through ROS/Ca^2^⁺-dependent signaling, remains unclear.^34,35^

Our results expand on prior work, which has mostly focused on the anti-inflammatory or antioxidant effects of APS, by providing direct mechanistic evidence linking APS to mitochondrial remodeling. Interestingly, APS has been shown to inhibit oxidative stress-induced injury in diabetic cardiomyopathy through suppression of NADPH oxidase and TGF-β signaling, suggesting that mitochondrial pathways may act in parallel with canonical stress and fibrosis pathways.^29^

Nonetheless, several limitations must be considered. First, all experiments were conducted *in vitro* using the HL-1 murine cardiomyocyte cell line. While HL-1 cells retain many characteristics of primary cardiomyocytes and are widely used in cardiovascular research, they are an immortalized cell line and may not fully replicate the complex metabolic, biomechanical, and neurohumoral environment of the *in vivo* myocardium. Therefore, validation of our findings in primary cardiomyocytes and, more importantly, in appropriate animal models of heart failure or mitochondrial dysfunction is essential before drawing definitive conclusions about the therapeutic potential of APS. Second, while we identified AMPK as a key mediator, the initial sensing mechanism by which APS triggers AMPK activation remains to be determined. Finally, the pharmacokinetics, tissue targeting, and long-term safety of APS have not been adequately evaluated. Given its polysaccharide nature, cellular uptake efficiency and metabolic stability in vivo require further investigation.

## Conclusion

In conclusion, our findings reveal that APS promotes mitochondrial biogenesis and improves respiratory function via activation of the AMPK/PGC-1α signaling pathway. This study provides new mechanistic insight into the mitochondrial-protective effects of APS and lays the groundwork for future translational studies in cardiac metabolic disorders. Validation of these findings in preclinical heart failure models and elucidation of upstream signaling will be critical for establishing APS as a viable therapeutic strategy.

## Supplemental Materials

Supplementary Materials For additional information, please click here.
